# Biguanidinium dichloride

**DOI:** 10.1107/S1600536808006144

**Published:** 2008-03-07

**Authors:** Gustavo Portalone

**Affiliations:** a Chemistry Department, ‘Sapienza’ University of Rome, P. le A. Moro, 5, I-00185 Rome, Italy

## Abstract

The asymmetric unit of the title compound, C_2_H_9_N_5_^2+^·2Cl^−^, is composed of one diprotonated biguanidinium cation and two chloride anions. The diprotonated cation consists of two planar halves twisted by 36.42 (6)°. The ions are associated in the crystal structure by extensive hydrogen bonding into a three-dimensional network; the diprotonated biguanidinium cation is hydrogen bonded to the chloride anions.

## Related literature

For a general approach to the use of multiple-hydrogen-bonding DNA/RNA nucleobases as potential supra­molecular reagents, see: Portalone & Colapietro (2004 ▶, 2007 ▶ and references therein). For related crystal structures, see: Ernst (1977 ▶); Pinkerton & Schwarzenbach (1978 ▶); Martin & Pinkerton (1996 ▶); Martin *et al.* (1996 ▶, 1997 ▶); Kurzer & Pitchfork (1968 ▶).
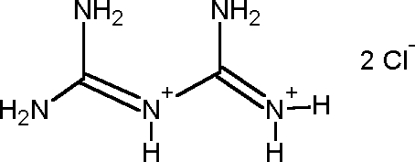


## Experimental

### 

#### Crystal data

C_2_H_9_N_5_^2+^·2Cl^−^
*M* *_r_* = 174.04
Monoclinic, 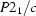

*a* = 6.43693 (9) Å
*b* = 16.93420 (18) Å
*c* = 6.65260 (8) Å
β = 98.6878 (12)°
*V* = 716.84 (1) Å^3^
*Z* = 4
Mo *K*α radiation
μ = 0.83 mm^−1^
*T* = 298 (2) K
0.20 × 0.20 × 0.15 mm

#### Data collection

Oxford Diffraction Xcalibur S CCD diffractometer
Absorption correction: multi-scan (*CrysAlis RED*; Oxford Diffraction, 2006 ▶) *T*_min_ = 0.852, *T*_max_ = 0.886
80724 measured reflections
2456 independent reflections
2349 reflections with *I* > 2σ(*I*)
*R*_int_ = 0.020

#### Refinement

*R*[*F* ^2^ > 2σ(*F* ^2^)] = 0.027
*wR*(*F* ^2^) = 0.070
*S* = 1.14
2456 reflections
119 parameters
All H-atom parameters refined
Δρ_max_ = 0.14 e Å^−3^
Δρ_min_ = −0.14 e Å^−3^

### 

Data collection: *CrysAlis CCD* (Oxford Diffraction, 2006 ▶; cell refinement: *CrysAlis RED* (Oxford Diffraction, 2006 ▶; data reduction: *CrysAlis RED*; program(s) used to solve structure: *SIR97* (Altomare *et al.*, 1999 ▶); program(s) used to refine structure: *SHELXL97* (Sheldrick, 2008 ▶); molecular graphics: *ORTEP-3* (Farrugia, 1997 ▶); software used to prepare material for publication: *WinGX* (Farrugia, 1999 ▶).

## Supplementary Material

Crystal structure: contains datablocks global, I. DOI: 10.1107/S1600536808006144/kp2161sup1.cif

Structure factors: contains datablocks I. DOI: 10.1107/S1600536808006144/kp2161Isup2.hkl

Additional supplementary materials:  crystallographic information; 3D view; checkCIF report

## Figures and Tables

**Table 1:** Hydrogen-bond geometry (Å, °)

*D*—H⋯*A*	*D*—H	H⋯*A*	*D*⋯*A*	*D*—H⋯*A*
N1—H1⋯Cl2	0.819 (18)	2.279 (18)	3.0796 (9)	166.0 (16)
N2—H21⋯Cl1^i^	0.833 (18)	2.530 (18)	3.2557 (10)	146.4 (15)
N2—H22⋯Cl2^ii^	0.852 (19)	2.34 (2)	3.1714 (10)	167.1 (17)
N3—H31⋯Cl2^i^	0.823 (19)	2.787 (19)	3.5098 (12)	147.8 (16)
N3—H32⋯Cl1^iii^	0.857 (19)	2.599 (19)	3.1933 (10)	127.4 (15)
N3—H32⋯Cl2	0.857 (19)	2.835 (19)	3.5454 (12)	141.3 (15)
N4—H41⋯Cl1	0.88 (2)	2.703 (19)	3.4178 (11)	139.4 (16)
N4—H42⋯Cl1^iv^	0.846 (19)	2.412 (19)	3.2295 (10)	162.8 (17)
N5—H51⋯Cl2^v^	0.853 (19)	2.413 (19)	3.2404 (12)	163.7 (16)
N5—H52⋯Cl1	0.874 (19)	2.369 (19)	3.1905 (11)	156.7 (17)

Symmetry codes: (i) 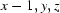; (ii) 
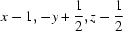
; (iii) 
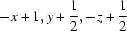
; (iv) 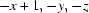; (v) 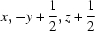.

**Fig. 1.**
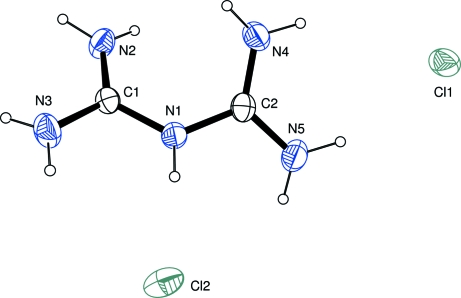
The molecular structure of (I) showing the atom-labelling scheme. Displacements ellipsoids are at the 50% probability level.

**Fig. 2.**
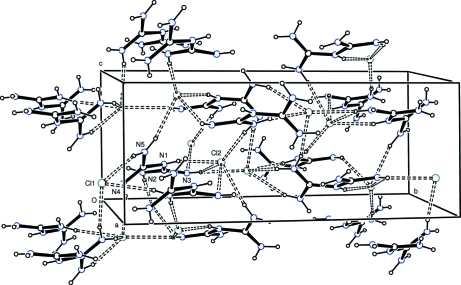
Crystal packing diagram for (I) viewed approximately down a. All atoms are shown as small spheres of arbitrary radii. Hydrogen bonding is indicated by dashed lines.

## supplementary crystallographic information

### Comment

Biguanidine derivatives, characterized by multiple hydrogen-bond donor sites, are good candidates to be coupled in the crystal with carefully selected molecules having multiple hydrogen-bond acceptor sites (Portalone & Colapietro, 2004, 2007). As a part of a more general study of multiple-hydrogen-bonding DNA/RNA nucleobases as potential supramolecular reagents, this paper reports the crystal structure of the title compound, (I), BIGH_2_^2+^2Cl^-^. The asymmetric unit of (I) (Fig. 1) consists of a diprotonated biguanidinium cation (BIGH_2_^2+^) and two chloride anions; protonation occurs at the bridge N atom and the imino N atom of the biguanidine molecule. The structure of the delocalized cation is very similar to those previously reported for the carbonate (BIGH_2_)CO_3_ and the sulfates (BIGH_2_)SO_4_.2H_2_O and (BIGH_2_)SO_4_.H_2_O (Pinkerton & Schwarzenbach, 1978), the dinitrate (BIGH_2_)NO_3_ (Martin *et al.*, 1996), the diperchlorate (BIGH_2_)2ClO_4_ (Martin & Pinkerton, 1996), the bis-dinitramide (BIGH_2_)(DN)_2_ and (BIGH_2_)(DN)_2_.H_2_O (Martin *et al.*, 1997). BIGH_2_^2+^ is composed of two planar halves sharing the atom N(1). These two planar parts are twisted with respect to each other by 36.42 (6)°. The C—N terminal bond lengths are shorter due to delocalization of π-electron density through the planar fragments. The lack of complete planarity of the cation is due to steric interaction between the hydrogen atoms. This interaction induces a strain in the molecule which is manifested by the opening of the angle at the bridging N atom [C1—N1—C2, 127.9 (1)°]. The weakening of the bridges bonds is due to the lowered basicity of the bridge N atom on protonation [pk_a_^I^ = 11.5; pk_a_^II^ = 2.9 (Kurzer & Pitchfork, 1968)] and is manifested by the longer C—N1 bridging bonds [1.363 (1) - 1.372 (1) Å] and the shorter terminal C—N bonds [1.306 (1) - 1.321 (1) Å], comparing with the corresponding ones reported for BIGH^+^Cl^-^ (Ernst, 1977). Analysis of the crystal packing of (I) (Fig. 2) shows that the structure is stabilized by ten hydrogen bonds N—H···Cl^-^ involving all protons (Table 1) which account for the relatively high density (D*_x_* = 1.61 Mg m^-3^).

### Experimental

Biguanide (0.1 mmol, Sigma Aldrich at 98% purity) was dissolved in water (9 ml) and heated under reflux for 2 h. After cooling a solution to an ambient temperature, while stirring, HCl (6 mol *L*^-1^) was added dropwise until the pH = 2. Crystals suitable for single-crystal X-ray diffraction were obtained by slow evaporation of the solvent after a few days.

### Refinement

All H atoms were found in a difference map and refined isotropically.

### Crystal data

**Table:**

C_2_H_9_N_5_^2+^·2Cl^–^	*F*_000_ = 360
*M**_r_* = 174.04	*D*_x_ = 1.613 Mg m^−^^3^
Monoclinic, *P*2_1_/*c*	Mo *K*α radiation λ = 0.71073 Å
Hall symbol: -P 2ybc	Cell parameters from 80724 reflections
*a* = 6.43693 (9) Å	θ = 3.2–32.0º
*b* = 16.93420 (18) Å	µ = 0.83 mm^−^^1^
*c* = 6.65260 (8) Å	*T* = 298 (2) K
β = 98.6878 (12)º	Plate, colourless
*V* = 716.841 (15) Å^3^	0.20 × 0.20 × 0.15 mm
*Z* = 4	

### Data collection

**Table:**

Oxford Diffraction Xcalibur S CCD diffractometer	2456 independent reflections
Radiation source: Enhance (Mo) X-ray source	2349 reflections with *I* > 2σ(*I*)
Monochromator: graphite	*R*_int_ = 0.020
Detector resolution: 16.0696 pixels mm^-1^	θ_max_ = 32.0º
*T* = 298(2) K	θ_min_ = 3.2º
ω and φ scans	*h* = −9→9
Absorption correction: multi-scan(CrysAlis RED; Oxford Diffraction, 2006)	*k* = −25→25
*T*_min_ = 0.852, *T*_max_ = 0.886	*l* = −9→9
80724 measured reflections	

### Refinement

**Table:**

Refinement on *F*^2^	Hydrogen site location: inferred from neighbouring sites
Least-squares matrix: full	All H-atom parameters refined
*R*[*F*^2^ > 2σ(*F*^2^)] = 0.027	*w* = 1/[σ^2^(*F*_o_^2^) + (0.0293*P*)^2^ + 0.2376*P*] where *P* = (*F*_o_^2^ + 2*F*_c_^2^)/3
*wR*(*F*^2^) = 0.071	(Δ/σ)_max_ < 0.001
*S* = 1.14	Δρ_max_ = 0.14 e Å^−^^3^
2456 reflections	Δρ_min_ = −0.14 e Å^−^^3^
119 parameters	Extinction correction: SHELXL97 (Sheldrick, 2008), Fc^*^=kFc[1+0.001xFc^2^λ^3^/sin(2θ)]^-1/4^
Primary atom site location: structure-invariant direct methods	Extinction coefficient: 0.110 (6)
Secondary atom site location: difference Fourier map	

### Special details

**Table:**

Geometry. All e.s.d.'s (except the e.s.d. in the dihedral angle between two l.s. planes) are estimated using the full covariance matrix. The cell e.s.d.'s are taken into account individually in the estimation of e.s.d.'s in distances, angles and torsion angles; correlations between e.s.d.'s in cell parameters are only used when they are defined by crystal symmetry. An approximate (isotropic) treatment of cell e.s.d.'s is used for estimating e.s.d.'s involving l.s. planes.
Refinement. Refinement of *F*^2^ against ALL reflections. The weighted *R*-factor *wR* and goodness of fit *S* are based on *F*^2^, conventional *R*-factors *R* are based on *F*, with *F* set to zero for negative *F*^2^. The threshold expression of *F*^2^ > σ(*F*^2^) is used only for calculating *R*-factors(gt) *etc*. and is not relevant to the choice of reflections for refinement. *R*-factors based on *F*^2^ are statistically about twice as large as those based on *F*, and *R*- factors based on ALL data will be even larger.

### Fractional atomic coordinates and isotropic or equivalent isotropic displacement parameters (Å^2^)

**Table:**

	*x*	*y*	*z*	*U*_iso_*/*U*_eq_	
Cl1	0.86502 (5)	−0.053995 (15)	0.27026 (4)	0.03288 (9)	
Cl2	0.62775 (5)	0.315420 (18)	0.37349 (5)	0.03605 (9)	
N1	0.35152 (13)	0.16618 (5)	0.32041 (15)	0.02556 (17)	
H1	0.430 (3)	0.2034 (10)	0.356 (3)	0.039 (4)*	
N2	0.00539 (15)	0.12772 (6)	0.20409 (16)	0.02955 (19)	
H21	0.025 (3)	0.0812 (11)	0.243 (3)	0.038 (4)*	
H22	−0.109 (3)	0.1408 (11)	0.130 (3)	0.048 (5)*	
N3	0.09938 (18)	0.25798 (6)	0.21118 (17)	0.0335 (2)	
H31	−0.025 (3)	0.2708 (11)	0.195 (3)	0.046 (5)*	
H32	0.193 (3)	0.2941 (11)	0.232 (3)	0.044 (5)*	
N4	0.38953 (17)	0.03968 (6)	0.18533 (15)	0.0304 (2)	
H41	0.461 (3)	−0.0045 (12)	0.196 (3)	0.050 (5)*	
H42	0.308 (3)	0.0513 (11)	0.078 (3)	0.047 (5)*	
N5	0.62338 (15)	0.08686 (7)	0.45410 (16)	0.0324 (2)	
H51	0.646 (3)	0.1178 (11)	0.556 (3)	0.044 (5)*	
H52	0.711 (3)	0.0485 (11)	0.440 (3)	0.051 (5)*	
C1	0.14797 (15)	0.18273 (5)	0.24248 (14)	0.02118 (17)	
C2	0.45468 (15)	0.09538 (6)	0.31785 (15)	0.02354 (18)	

### Atomic displacement parameters (Å^2^)

**Table:**

	*U*^11^	*U*^22^	*U*^33^	*U*^12^	*U*^13^	*U*^23^
Cl1	0.04007 (15)	0.02213 (13)	0.03406 (15)	0.00402 (9)	−0.00208 (10)	−0.00296 (9)
Cl2	0.03243 (14)	0.03940 (16)	0.03375 (15)	−0.01269 (10)	−0.00333 (10)	0.00083 (10)
N1	0.0205 (4)	0.0213 (4)	0.0344 (4)	−0.0012 (3)	0.0024 (3)	−0.0021 (3)
N2	0.0220 (4)	0.0256 (4)	0.0390 (5)	−0.0017 (3)	−0.0020 (3)	0.0058 (4)
N3	0.0368 (5)	0.0215 (4)	0.0420 (6)	0.0052 (4)	0.0055 (4)	0.0047 (4)
N4	0.0379 (5)	0.0269 (4)	0.0257 (4)	0.0089 (4)	0.0022 (4)	−0.0017 (3)
N5	0.0253 (4)	0.0399 (5)	0.0312 (5)	0.0067 (4)	0.0010 (3)	0.0010 (4)
C1	0.0229 (4)	0.0203 (4)	0.0208 (4)	0.0012 (3)	0.0047 (3)	0.0022 (3)
C2	0.0218 (4)	0.0261 (4)	0.0238 (4)	0.0022 (3)	0.0068 (3)	0.0031 (3)

### Geometric parameters (Å, °)

**Table:**

N1—C1	1.3634 (13)	N3—H32	0.857 (19)
N1—C2	1.3719 (13)	N4—C2	1.3156 (14)
N1—H1	0.819 (18)	N4—H41	0.88 (2)
N2—C1	1.3056 (13)	N4—H42	0.846 (19)
N2—H21	0.833 (18)	N5—C2	1.3131 (14)
N2—H22	0.852 (19)	N5—H51	0.853 (19)
N3—C1	1.3211 (13)	N5—H52	0.874 (19)
N3—H31	0.823 (19)		
			
C1—N1—C2	127.89 (9)	H41—N4—H42	121.2 (17)
C1—N1—H1	117.6 (12)	C2—N5—H51	120.4 (12)
C2—N1—H1	113.6 (12)	C2—N5—H52	119.2 (13)
C1—N2—H21	122.9 (12)	H51—N5—H52	120.4 (17)
C1—N2—H22	116.6 (13)	N2—C1—N3	120.97 (10)
H21—N2—H22	120.4 (17)	N2—C1—N1	122.31 (9)
C1—N3—H31	118.5 (13)	N3—C1—N1	116.70 (9)
C1—N3—H32	121.3 (12)	N5—C2—N4	122.04 (10)
H31—N3—H32	119.0 (17)	N5—C2—N1	116.03 (10)
C2—N4—H41	116.7 (12)	N4—C2—N1	121.93 (9)
C2—N4—H42	119.7 (12)		
			
C2—N1—C1—N2	19.96 (17)	C1—N1—C2—N5	−159.12 (10)
C2—N1—C1—N3	−161.49 (10)	C1—N1—C2—N4	21.55 (17)

### Hydrogen-bond geometry (Å, °)

**Table:**

*D*—H···*A*	*D*—H	H···*A*	*D*···*A*	*D*—H···*A*
N1—H1···Cl2	0.819 (18)	2.279 (18)	3.0796 (9)	166.0 (16)
N2—H21···Cl1^i^	0.833 (18)	2.530 (18)	3.2557 (10)	146.4 (15)
N2—H22···Cl2^ii^	0.852 (19)	2.34 (2)	3.1714 (10)	167.1 (17)
N3—H31···Cl2^i^	0.823 (19)	2.787 (19)	3.5098 (12)	147.8 (16)
N3—H32···Cl1^iii^	0.857 (19)	2.599 (19)	3.1933 (10)	127.4 (15)
N3—H32···Cl2	0.857 (19)	2.835 (19)	3.5454 (12)	141.3 (15)
N4—H41···Cl1	0.88 (2)	2.703 (19)	3.4178 (11)	139.4 (16)
N4—H42···Cl1^iv^	0.846 (19)	2.412 (19)	3.2295 (10)	162.8 (17)
N5—H51···Cl2^v^	0.853 (19)	2.413 (19)	3.2404 (12)	163.7 (16)
N5—H52···Cl1	0.874 (19)	2.369 (19)	3.1905 (11)	156.7 (17)

Symmetry codes: (i) *x*−1, *y*, *z*; (ii) *x*−1, −*y*+1/2, *z*−1/2; (iii) −*x*+1, *y*+1/2, −*z*+1/2; (iv) −*x*+1, −*y*, −*z*; (v) *x*, −*y*+1/2, *z*+1/2.
